# Supplementary material for: A novel code representation for detecting Java code clones using high-level and abstract compiled code representations
Source: PLoS One. 2024 May 10;19(5):e0302333. doi: 10.1371/journal.pone.0302333 (PMC11086904; doi:10.1371/journal.pone.0302333)
Supplement: S1 File — (PDF) [file pone.0302333.s001.pdf]

A novel code representation for detecting Java code clones using high-level and abstract compiled code representations

Fahmi H. Quradaa [1,2*], Sara Shahzad[1], Rashad Saeed[1,2], Mubarak M. Sufyan[1,3]

1: Department of Computer Science, University of Peshawar, Peshawar, Pakistan

2: Department of Computer Science, Aden Community College, Aden, Yemen

3: Department of Networks and Cyber Security, AlJanad University Of Science and Technology, Taiz, Yemen

1. Abstract Syntax Tree (AST) Features

Table 1. AST features

	Name	Example
1	No. of BlockStmt	{ … }
2	No. of BreakStmt	break
3	No. of CastExpr	(long)15
4	No. of CatchClause	catch (Exception e) { … }
5	No. of CharLiteralExpr	‘a’
6	No. of ClassExpr	Object.class
7	No. of AssignExpr	a=5
8	No. of BinaryExpr	a && b
9	No. of ArrayAccessExpr	getNames()[15*15]
10	No. of ArrayCreationExpr	new int[5]
11	No. of ArrayCreationLevel	new int[1][2]
12	No. of ArrayInitializerExpr	new int[][] {{1, 1}, {2, 2}}
13	No. of ClassOrInterfaceType	Object<br>HashMap<String, String><br>java.util.Punchcard
14	No. of ConditionalExpr	if(a)
15	No. of ContinueStmt	continue
16	No. of DoStmt	do { … } while (a==0)
17	No. of DoubleLiteralExpr	100.1f
18	No. of EnclosedExpr	(1+1)
19	No. of EnumConstantDeclaration	X { A(1), B(2) }
20	No. of EnumDeclaration	enum X { … }
21	No. of ExplicitConstructorInvocationStmt	class X { X() { super(15); } }<br>class X { X() { this(1, 2); } }
22	No. of ExpressionStmt	Wraps Expressions into Statements
23	No. of FieldAccessExpr	person.name
24	No. of FieldDeclaration	private static int a=15
25	No. of ForeachStmt	for(Object o: objects) { … }
26	No. of ForStmt	for(int a=3,b=5; a<99; a++) { … }
27	No. of IfStmt	if(a==5) hurray() else boo()
28	No. of InitializerDeclaration	class X { static { a=3; } }
29	No. of InstanceOfExpr	tool instanceof Drill
30	No. of IntegerLiteralExpr	8934
31	No. of IntersectionType	Serializable & Cloneable
32	No. of LambdaExpr	(a, b) -> a+b
33	No. of LongLiteralExpr	8934l
34	No. of MemberValuePair	@Counters(a=15)
35	No. of MarkerAnnotationExpr	@Override
36	No. of MethodCallExpr	circle.circumference()
37	No. of MethodDeclaration	public int abc() {return 1;}
38	No. of MethodReferenceExpr	System.out::println
39	No. of Name	it.may.contain.dots
40	No. of NameExpr	int x = a + 3

41	No. of NullLiteralExpr	null
42	No. of ObjectCreationExpr	new HashMap.Entry(15)
43	No. of Parameter	int abc(String x)
44	No. of PrimitiveType	int
45	No. of ReturnStmt	return 5 * 5
46	No. of StringLiteralExpr	“Hello World!”
47	No. of SuperExpr	super
48	No. of SwitchEntryStmt	case 1:
49	No. of SwitchStmt	switch(a) { … }
50	No. of SynchronizedStmt	synchronized (a123) { … }
51	No. of ThisExpr	this
52	No. of ThrowStmt	throw new Exception()
53	No. of TryStmt	try (…) { … } catch (…) { } finally { }
54	No. of TypeExpr	World::greet
55	No. of TypeParameter	<U> U getU() { … }
56	No. of UnaryExpr	1++
57	No. of UnionType	catch(IOException NullPointerException ex)
58	No. of UnknownType	DoubleToIntFunction d = x -> (int)x + 1
59	No. of VariableDeclarationExpr	final int x=3, y=55
60	No. of VariableDeclarator	int x = 14
61	No. of VoidType	void helloWorld() { … }
62	No. of WhileStmt	while(true) { … }
63	No. of WildcardType	Collection<?> c

2. Baf Intermediate Representation Instructions

Table 2. BAF IR Features

	**Instruction**	**Description**
1	No. of Load	Load variable from local variable
2	No. of Store	Store variable into local variable
3	No. of Inc	Increment local variable by constant
4	No. of fieldget	Fetch field from object
5	No. of fieldput	Set field in object
6	No. of staticget	Get static field from class
7	No. of staticput	Set static field in class
8	No. of virtualinvoke	Invoke instance method; dispatch based on class
9	No. of specialinvoke	Invoke instance method; direct invocation of instance initialization methods and methods of the current class and its supertypes
10	No. of staticinvoke	Invoke a class (static) method
11	No. of interfaceinvoke	Invoke interface method
12	No. of Dup1	Duplicate the top operand stack value
13	No. of Dup2	Duplicate the top one or two operand stack values
14	No. of New	Create new object
15	No. of Ifne	Jump if value1 <> value 2
16	No. of Ifeq	Jump if value 1 = value 2
17	No. of Ifge	Jump if value 1 >= value 2
18	No. of Ifle	Jump if value 1 <= value 2
19	No. of Ifgt	Jump if value 1 > value 2
20	No. of Iflt	Jump if value 1 < value 2
21	No. of Pop	Pop the top operand stack value
22	No. of Push	Push variable into stack
23	No. of Lookupswitch	Access jump table by key match and jump
24	No. of Tableswitch	Access jump table by index and jump
25	No. of Return	Return value from method
26	No. of Ifcmpne	Branch if and only if Value1 <> value 2
27	No. of Ifcmpeq	Branch if and only if Value1 = value 2
28	No. of Ifcmpge	Branch if and only if value1 >= value2
29	No. of Ifcmple	Branch if and only if Value1 <= value 2
30	No. of Ifcmpgt	Branch if and only if value1 > value2
31	No. of Ifcmplt	Branch if and only if value1 < value2

32	No. of ifnull	Check if contains null
33	No. of Cmpg	Compare value1 with value2 if value1 > value2 , value 1 pushed onto operand stack. otherwise, push 0 onto stack.
34	No. of Cmp	Compare value1 with value2 if value1 > value2 , value 1 pushed onto operand stack.<br>Otherwise if value1 < value2 then push -1 onto stack, otherwise, push 0 onto stack as value1 = value 2.
35	No. of Cmpl	Compare value1 with value2 if value1 < value2 , value 1 pushed onto operand stack. otherwise, push 0 onto stack.
36	No. of sub	Subtract two varaible
37	No. of Add	Add two variables
38	No. of div	Divide value1 by value2 that pops from stack
39	No. of Mul	Multiply value1 with value2 that pops from stack
40	No. of Rem	Remainder of division operation
41	No. of Neg	Negate variable. If value is positive it will be converted into -value
42	No. of Instanceof	instanceof determines whether objectref is an instance of T
43	No. of Shl	Arithmetic shift left
44	No. of Shr	Arithmetic shift right
45	No. of Ushr	Logical shift right
46	No. of Goto	Branch always
47	No. of Throw	Throw exception or error
48	No. of Checkcast	Check whether object is of given type
49	No. of Newarray	Create new array
50	No. of Newmultiarray	Create new multidimensional array
51	No. of Arraylength	Get length of array
52	No. of arraywrite	Write an array
53	No. of arrayread	Read an array
54	No. of Entermonitor	Enter monitor for object
55	No. of Exitmonitor	Exit monitor for object
56	No. of Nop	Do nothing
57	No. of Breakpoint	Set a breakpoint at the instruction
58	No. of And	Boolean AND
59	No. of Or	Boolean OR
60	No. of Xor	Boolean XOR

3. Jimple Intermediate Representation and Jimple Block Program Dependency Graph Features

Table 3. Jimple IR Features

Feature category	Feature Name	Feature Description
Core statement	No. of NopStmt	no operation
	No. of DefinitionStmt	
	No. of IdentityStmt	identityStmts are statements which define locals to be pre-loaded with special values such as parameters or the this value.
	No. of AssignStmt	The assignStmt statement is the most used JIMPLE instruction. It has four forms: assigning a r value to a local, or an immediate (a local or a constant) to a static field, to an instance field or to an array reference.
Statements for intra-procedural control-flow	No. of IfStmt	represent conditional jump
	No. of GotoStmt	represent unconditional jump
	No. of TableSwitchStmt	switchStmt can either be a lookupswitch or a tableswitch. The lookupswitch takes a set of integers values, whereas tableswitch takes a range of integer values for the lookup values, as the tableswitch and lookupswitch Java bytecodes do. The target destinations are specified by labels.
	No. of LookupSwitchStmt	
	No. of breakpointStmt	represent the breakpoint

Statements for intre-procedural control-flow	No. of InvokeStmt	invokeStmt represents an invoke without an assignment to a local.
	No. of ReturnStmt	returnStmt can either represent a return void, or a return of a value, specified by a local or a constant.
	No. of ReturnVoidStmt	
Monitor Statements	No. of EnterMonitorStmt	monitorStmt represents the enter/exitmonitor bytecodes. They take a local or constant as the monitor lock.
	No. of ExitMonitorStmt	
	No. of MonitorStmt	
	No. of ThrowStmt	throwStmt represents the explicit throwing of an exception.
	No. of RetStmt	Not used, returns from a JSR

Table 4. Jimple Block Program Dependency Graph Features

	Category	Feature Description
1	Control Flow Graph	Number of nodes in the CFG
2		Number of Edges in the CFG
3		Cyclomatic complexity of CFG = edges-nodes +2
4		Number of nodes have greater than 1 predecessors
5		Number of nodes have 1 predecessor
6		Number of nodes have greater than 1 successors
7		Number of nodes have 1 successor
8	Block Control Flow Graph	Number of blocks in Block CFG
9		Number of blocks have greater than 1 blocks predecessors
10		Number of blocks have 1 block predecessor
11		Number of blocks have greater than 1 blocks successors
12		Number of blocks have 1 block successor
13		Maximum Block (LOC)
14		Minimum Block (LOC)
15		Total length of the method (LOC)
16		Percentage of LOC in all Blocks
17	Program Dependency Graph	Number of PDG region
18		Number of strong regions in PDG
19		Number of weak regions in PDG
20		Number of region node in PDG
21		Number of PDGNODE node in PDG
22		Number of dependency Edges in PDG
23		Number of control flow edges in PDG
24		Number of dependency-back edges in PDG
25		Number of dependency edges between Region node and Region node in PDG
26		Number of dependency edges between Region node and Block node in PDG
27		Number of dependency edges between Block node and Region node in PDG
28		Number of dependency edges between Block node and Block node in PDG
29		Number of control flow edges between Region node and Region node in PDG
30		Number of control flow edges between Region node and Block node in PDG
31		Number of control flow edges between Block node and Region node in PDG
32		Number of control flow edges between Block node and Block node in PDG
33		Number of dependency-back edges between Region node and Region node in PDG
34		Number of dependency-back edges between Region node and Block node in PDG
35		Number of dependency-back edges between Block node and Region node in PDG
36		Number of dependency-back edges between Block node and Block node in PDG
