# Supplementary material for: A novel code representation for detecting Java code clones using high-level and abstract compiled code representations
Source: PLoS One. 2024 May 10;19(5):e0302333. doi: 10.1371/journal.pone.0302333 (PMC11086904; doi:10.1371/journal.pone.0302333)
Supplement: S3 File — (PDF) [file pone.0302333.s003.pdf]

# A novel code representation for detecting Java code clones using high-level and abstract compiled code representations

Fahmi H. Quradaa [1,2*], Sara Shahzad[1], Rashad Saeed[1,2], Mubarak M. Sufyan[1,3]

1: Department of Computer Science, University of Peshawar, Peshawar, Pakistan

2: Department of Computer Science, Aden Community College, Aden, Yemen

3: Department of Networks and Cyber Security, AlJanad University Of Science and Technology, Taiz, Yemen

## 1. Evaluation of Performance of Various Classifiers: Experimental Results

| | Classifiers | Combination technique | Accuracy | Precision | Recall | F1 |
|---|---|---|---|---|---|---|
| 1 | Logistic Regression | Linear Combined features | 0.886541 | 0.89419403 | 0.878477 | 0.886266 |
| 2 | Naive Bayes | | 0.665908 | 0.73515083 | 0.591528 | 0.657536 |
| 3 | RandomForest | | 0.957143 | 0.95789765 | 0.956389 | 0.957142 |
| 4 | RotationForest | | 0.962731 | 0.96334034 | 0.962132 | 0.962735 |
| 5 | SVM | | 0.878041 | 0.93223417 | 0.819407 | 0.872295 |
| 6 | LDA | | 0.733726 | 0.74914734 | 0.717644 | 0.733131 |
| 7 | LogitBoost | | 0.945715 | 0.94743687 | 0.943996 | 0.945712 |
| 8 | J48 | | 0.894517 | 0.9336685 | 0.854041 | 0.892165 |
| 9 | Bagging | | 0.927002 | 0.93625214 | 0.917476 | 0.92677 |
| 10 | Random subspace | | 0.93585 | 0.94496766 | 0.926647 | 0.935705 |
| 11 | XGBClassifier | | 0.967395 | 0.96853364 | 0.966253 | 0.967391 |
| 12 | LightGBM | | 0.967563 | 0.96844997 | 0.966671 | 0.967559 |
| 13 | CatBoostClassifier | | 0.955379 | 0.95792362 | 0.952823 | 0.955366 |
| 14 | Random committee | | 0.946378 | 0.94706995 | 0.945678 | 0.946373 |
| 15 | FFNN | | 0.922211 | 0.92438599 | 0.920017 | 0.922192 |
| 1 | Logistic Regression | Multiplication Combined Features | 0.867551 | 0.885973 | 0.847785858 | 0.866486 |
| 2 | Naive Bayes | | 0.645439 | 0.755224 | 0.532328041 | 0.629531 |
| 3 | RandomForest | | 0.944942 | 0.955674 | 0.934101933 | 0.944765 |
| 4 | RotationForest | | 0.951749 | 0.958866 | 0.944526315 | 0.95164 |
| 5 | SVM | | 0.631255 | 0.860403 | 0.337734539 | 0.490422 |
| 6 | LDA | | 0.709644 | 0.755979 | 0.661993522 | 0.706617 |
| 7 | LogitBoost | | 0.929237 | 0.942486 | 0.915786126 | 0.928947 |
| 8 | J48 | | 0.832859 | 0.935546 | 0.728353365 | 0.818632 |
| 9 | Bagging | | 0.905214 | 0.938956 | 0.870706011 | 0.903605 |
| 10 | Random subspace | | 0.903844 | 0.937254 | 0.869567317 | 0.902186 |
| 11 | XGBClassifier | | 0.95806 | 0.965417 | 0.950619472 | 0.95796 |
| 12 | LightGBM | | 0.956228 | 0.965187 | 0.947184866 | 0.9561 |
| 13 | CatBoostClassifier | | 0.938002 | 0.954344 | 0.921469393 | 0.937625 |
| 14 | Random committee | | 0.93285 | 0.944415 | 0.921137747 | 0.932635 |
| 15 | FFNN | | 0.91238 | 0.915288 | 0.909480371 | 0.912333 |
| 1 | Logistic Regression | Distance Combined Features | 0.801404924 | 0.819541 | 0.778741 | 0.798629 |
| 2 | Naive Bayes | | 0.548951349 | 0.52823 | 0.589149 | 0.557467 |
| 3 | RandomForest | | 0.916717923 | 0.941007 | 0.891512 | 0.915607 |
| 4 | RotationForest | | 0.921759936 | 0.940049 | 0.902529 | 0.920903 |
| 5 | SVM | | 0.807459037 | 0.852765 | 0.748371 | 0.797229 |
| 6 | LDA | | 0.667798504 | 0.68986 | 0.641591 | 0.665044 |
| 7 | LogitBoost | | 0.896256617 | 0.914365 | 0.876789 | 0.895184 |
| 8 | J48 | | 0.826254096 | 0.812072 | 0.846178 | 0.828665 |
| 9 | Bagging | | 0.903382069 | 0.923277 | 0.882369 | 0.90237 |
| 10 | Random subspace | | 0.83690026 | 0.896849 | 0.76984 | 0.828696 |
| 11 | XGBClassifier | | 0.937649777 | 0.950424 | 0.924375 | 0.937212 |
| 12 | LightGBM | | 0.930969666 | 0.946196 | 0.915006 | 0.930333 |
| 13 | CatBoostClassifier | | 0.907945551 | 0.927564 | 0.886985 | 0.906818 |
| 14 | Random committee | | 0.899752122 | 0.921363 | 0.87685 | 0.898568 |
| 15 | FFNN | | 0.878021175 | 0.890891 | 0.864107 | 0.877284 |

## 2. Evaluation of Performance with Different Dataset Sizes and Feature Types: Experimental Results.

| Dataset Size | Combination technique | Feature type | classifiers | Accuracy | Precision | Recall | F1 |
|---|---|---|---|---|---|---|---|
| 10,000 | Linear | AST+BAF+Jimple | RandomForest | 0.963 | 0.96419228 | 0.9618 | 0.96299015 |
| | | | RotationForest | 0.9643 | 0.96519598 | 0.9634 | 0.96429589 |
| | | | XGBClassifier | 0.9639 | 0.96618587 | 0.9616 | 0.96388262 |
| | | | LightGBM | 0.9642 | 0.96560264 | 0.9628 | 0.96419033 |
| | | | FFNN | 0.9343 | 0.93689374 | 0.9318 | 0.93427632 |
| | | AST | RandomForest | 0.91644 | 0.917043 | 0.91584 | 0.916436 |
| | | | RotationForest | 0.9326 | 0.931836 | 0.9334 | 0.93261 |
| | | | XGBClassifier | 0.9239 | 0.926584 | 0.9212 | 0.923882 |
| | | | LightGBM | 0.9335 | 0.935383 | 0.9316 | 0.933481 |
| | | | FFNN | 0.88516 | 0.885209 | 0.88516 | 0.885171 |
| | | BAF | RandomForest | 0.9121 | 0.914177 | 0.91 | 0.912081 |
| | | | RotationForest | 0.9221 | 0.922998 | 0.9212 | 0.922092 |
| | | | XGBClassifier | 0.9116 | 0.914775 | 0.9084 | 0.91157 |
| | | | LightGBM | 0.9135 | 0.915587 | 0.9114 | 0.913483 |
| | | | FFNN | 0.8617 | 0.867327 | 0.856 | 0.861599 |
| | | Jimple | RandomForest | 0.87577 | 0.879605 | 0.87187 | 0.875714 |
| | | | RotationForest | 0.9193 | 0.921369 | 0.9172 | 0.919269 |
| | | | XGBClassifier | 0.8905 | 0.894179 | 0.8868 | 0.890461 |
| | | | LightGBM | 0.89595 | 0.899808 | 0.89205 | 0.895905 |
| | | | FFNN | 0.85256 | 0.857524 | 0.84836 | 0.852643 |
| | Distance | AST+BAF+Jimple | RandomForest | 0.9108 | 0.944832 | 0.875 | 0.908586 |
| | | | RotationForest | 0.9134 | 0.93624 | 0.889 | 0.911984 |
| | | | XGBClassifier | 0.9243 | 0.94143 | 0.9062 | 0.923443 |
| | | | LightGBM | 0.9241 | 0.94479 | 0.9024 | 0.923066 |
| | | | FFNN | 0.894 | 0.907143 | 0.8796 | 0.892915 |
| | | AST | RandomForest | 0.8562 | 0.937598 | 0.7692 | 0.845276 |
| | | | RotationForest | 0.8571 | 0.896385 | 0.811 | 0.851593 |
| | | | XGBClassifier | 0.905668 | 0.8316 | 0.867035 | 0.905668 |
| | | | LightGBM | 0.908351 | 0.8226 | 0.863368 | 0.908351 |
| | | | FFNN | 0.8441 | 0.8511 | 0.8356 | 0.843025 |
| | | BAF | RandomForest | 0.8466 | 0.914973 | 0.7698 | 0.836157 |
| | | | RotationForest | 0.8449 | 0.884927 | 0.7968 | 0.838498 |
| | | | XGBClassifier | 0.8524 | 0.885181 | 0.8134 | 0.847634 |
| | | | LightGBM | 0.8513 | 0.890233 | 0.805 | 0.845442 |
| | | | FFNN | 0.8422 | 0.859183 | 0.820565 | 0.839129 |
| | | Jimple | RandomForest | 0.8961 | 0.920093 | 0.8696 | 0.89409 |
| | | | RotationForest | 0.8903 | 0.909971 | 0.868 | 0.888479 |
| | | | XGBClassifier | 0.8992 | 0.917669 | 0.8786 | 0.897706 |
| | | | LightGBM | 0.8988 | 0.920629 | 0.8746 | 0.897013 |
| | | | FFNN | 0.869633 | 0.902335 | 0.832646 | 0.865691 |
| | Multiplicative | AST+BAF+Jimple | RandomForest | 0.9449 | 0.962134 | 0.9274 | 0.944446 |
| | | | RotationForest | 0.946 | 0.958516 | 0.9332 | 0.94568 |
| | | | XGBClassifier | 0.9502 | 0.962401 | 0.9378 | 0.949939 |
| | | | LightGBM | 0.9505 | 0.962035 | 0.9388 | 0.950264 |
| | | | FFNN | 0.893 | 0.946185 | 0.8374 | 0.888374 |
| | | AST | RandomForest | 0.9329 | 0.95946 | 0.9058 | 0.931847 |
| | | | RotationForest | 0.9301 | 0.956069 | 0.9034 | 0.928984 |
| | | | XGBClassifier | 0.9376 | 0.957693 | 0.917 | 0.936899 |
| | | | LightGBM | 0.935 | 0.957221 | 0.9122 | 0.934159 |
| | | | FFNN | 0.861 | 0.933743 | 0.9014 | 0.917085 |
| | | BAF | RandomForest | 0.9249 | 0.947821 | 0.901 | 0.923783 |
| | | | RotationForest | 0.9196 | 0.935679 | 0.9024 | 0.918702 |
| | | | XGBClassifier | 0.927 | 0.943517 | 0.9096 | 0.926238 |
| | | | LightGBM | 0.9248 | 0.9452 | 0.9034 | 0.923793 |
| | | | FFNN | 0.8774 | 0.924777 | 0.8808 | 0.902144 |
| | | Jimple | RandomForest | 0.9245 | 0.948972 | 0.899 | 0.923271 |
| | | | RotationForest | 0.9248 | 0.939679 | 0.909 | 0.924043 |
| | | | XGBClassifier | 0.9301 | 0.948625 | 0.9108 | 0.929321 |
| | | | LightGBM | 0.9297 | 0.95061 | 0.908 | 0.928802 |

| | | | | | | | |
|---|---|---|---|---|---|---|---|
| | | | FFNN | 0.7518 | 0.877892 | 0.6124 | 0.704049 |
| 20,000 | Linear | AST+BAF+Jimple | RandomForest | 0.9667 | 0.96749729 | 0.9659 | 0.966697 |
| | | | RotationForest | 0.9671 | 0.96750177 | 0.9667 | 0.967098 |
| | | | XGBClassifier | 0.96695 | 0.96819868 | 0.9657 | 0.966945 |
| | | | LightGBM | 0.96705 | 0.96809599 | 0.966 | 0.967047 |
| | | | FFNN | 0.9382 | 0.94075142 | 0.9357 | 0.938179 |
| | | AST | RandomForest | 0.92685 | 0.927099 | 0.9266 | 0.926849 |
| | | | RotationForest | 0.943675 | 0.943828 | 0.943525 | 0.943674 |
| | | | XGBClassifier | 0.94565 | 0.947147 | 0.94415 | 0.945645 |
| | | | LightGBM | 0.94645 | 0.948297 | 0.9446 | 0.946443 |
| | | | FFNN | 0.89593 | 0.897844 | 0.89403 | 0.895913 |
| | | BAF | RandomForest | 0.936585 | 0.937928 | 0.935235 | 0.936579 |
| | | | RotationForest | 0.931565 | 0.931716 | 0.931415 | 0.931564 |
| | | | XGBClassifier | 0.94655 | 0.948341 | 0.94475 | 0.946541 |
| | | | LightGBM | 0.93545 | 0.937791 | 0.9331 | 0.935439 |
| | | | FFNN | 0.8873 | 0.889582 | 0.8851 | 0.887297 |
| | | Jimple | RandomForest | 0.89642 | 0.898806 | 0.89402 | 0.896406 |
| | | | RotationForest | 0.916435 | 0.91738 | 0.915485 | 0.916428 |
| | | | XGBClassifier | 0.9121 | 0.914738 | 0.90945 | 0.912084 |
| | | | LightGBM | 0.9243 | 0.926793 | 0.9218 | 0.924283 |
| | | | FFNN | 0.8606 | 0.867204 | 0.8541 | 0.860489 |
| | Distance | AST+BAF+Jimple | RandomForest | 0.92185 | 0.949026 | 0.8935 | 0.920431 |
| | | | RotationForest | 0.92155 | 0.939668 | 0.9023 | 0.920587 |
| | | | XGBClassifier | 0.93505 | 0.949311 | 0.9202 | 0.934522 |
| | | | LightGBM | 0.9305 | 0.945001 | 0.9153 | 0.929869 |
| | | | FFNN | 0.91105 | 0.92373 | 0.8972 | 0.910229 |
| | | AST | RandomForest | 0.863 | 0.938891 | 0.7821 | 0.85356 |
| | | | RotationForest | 0.86215 | 0.902539 | 0.8155 | 0.856857 |
| | | | XGBClassifier | 0.87755 | 0.914789 | 0.8357 | 0.873511 |
| | | | LightGBM | 0.8716 | 0.917625 | 0.8202 | 0.866244 |
| | | | FFNN | 0.85845 | 0.87074 | 0.8442 | 0.856854 |
| | | BAF | RandomForest | 0.8645 | 0.925455 | 0.7976 | 0.856903 |
| | | | RotationForest | 0.86155 | 0.899191 | 0.8178 | 0.856499 |
| | | | XGBClassifier | 0.86975 | 0.90067 | 0.834 | 0.866029 |
| | | | LightGBM | 0.86285 | 0.903867 | 0.8156 | 0.85752 |
| | | | FFNN | 0.85615 | 0.871374 | 0.8373 | 0.853948 |
| | | Jimple | RandomForest | 0.908 | 0.929746 | 0.8844 | 0.906487 |
| | | | RotationForest | 0.90575 | 0.927132 | 0.8824 | 0.90421 |
| | | | XGBClassifier | 0.9121 | 0.927864 | 0.8949 | 0.911071 |
| | | | LightGBM | 0.90575 | 0.922462 | 0.8873 | 0.904531 |
| | | | FFNN | 0.883 | 0.915244 | 0.8471 | 0.879704 |
| | Multiplicative | AST+BAF+Jimple | RandomForest | 0.9508 | 0.964762 | 0.9367 | 0.950511 |
| | | | RotationForest | 0.95325 | 0.96257 | 0.9438 | 0.953087 |
| | | | XGBClassifier | 0.95605 | 0.965215 | 0.9468 | 0.955917 |
| | | | LightGBM | 0.95475 | 0.965606 | 0.9438 | 0.95458 |
| | | | FFNN | 0.878 | 0.927468 | 0.8256 | 0.871687 |
| | | AST | RandomForest | 0.93935 | 0.96391 | 0.9145 | 0.938561 |
| | | | RotationForest | 0.9385 | 0.957721 | 0.9188 | 0.93785 |
| | | | XGBClassifier | 0.94425 | 0.959891 | 0.9283 | 0.943832 |
| | | | LightGBM | 0.94275 | 0.962093 | 0.9231 | 0.942199 |
| | | | FFNN | 0.83235 | 0.94335 | 0.9003 | 0.921272 |
| | | BAF | RandomForest | 0.92985 | 0.949005 | 0.9099 | 0.929023 |
| | | | RotationForest | 0.9243 | 0.938178 | 0.9095 | 0.923602 |
| | | | XGBClassifier | 0.86975 | 0.90067 | 0.834 | 0.866029 |
| | | | LightGBM | 0.86285 | 0.903867 | 0.8156 | 0.85752 |
| | | | FFNN | 0.85615 | 0.871374 | 0.8373 | 0.853948 |
| | | Jimple | RandomForest | 0.93225 | 0.953597 | 0.9102 | 0.931393 |
| | | | RotationForest | 0.93395 | 0.947321 | 0.92 | 0.933435 |
| | | | XGBClassifier | 0.93765 | 0.952684 | 0.9221 | 0.93713 |
| | | | LightGBM | 0.93495 | 0.954017 | 0.9153 | 0.934225 |
| | | | FFNN | 0.78795 | 0.844271 | 0.7748 | 0.792505 |
| 30,000 | Linear | AST+BAF+Jimple | RandomForest | 0.9669 | 0.96773123 | 0.966067 | 0.966897 |
| | | | RotationForest | 0.967567 | 0.9681315 | 0.967 | 0.967565 |
| | | | XGBClassifier | 0.967433 | 0.96839941 | 0.966467 | 0.967431 |
| | | | LightGBM | 0.967267 | 0.96853102 | 0.966 | 0.967263 |
| | | | FFNN | 0.9381 | 0.93781203 | 0.938733 | 0.938178 |
| | | AST | RandomForest | 0.91671 | 0.917143 | 0.916277 | 0.916708 |

| | | | | | | | |
|---|---|---|---|---|---|---|---|
| | | | RotationForest | 0.922133 | 0.922467 | 0.9218 | 0.922132 |
| | | | XGBClassifier | 0.93 | 0.931296 | 0.9287 | 0.929996 |
| | | | LightGBM | 0.932667 | 0.933996 | 0.931333 | 0.932662 |
| | | | FFNN | 0.893333 | 0.896771 | 0.889867 | 0.8933 |
| | | BAF | RandomForest | 0.926627 | 0.928154 | 0.925093 | 0.926621 |
| | | | RotationForest | 0.926647 | 0.927975 | 0.925313 | 0.926642 |
| | | | XGBClassifier | 0.933333 | 0.935694 | 0.930967 | 0.933323 |
| | | | LightGBM | 0.934667 | 0.937227 | 0.9321 | 0.934656 |
| | | | FFNN | 0.886667 | 0.890173 | 0.883133 | 0.886633 |
| | | Jimple | RandomForest | 0.9236 | 0.925983 | 0.9212 | 0.923585 |
| | | | RotationForest | 0.891437 | 0.892827 | 0.890037 | 0.891429 |
| | | | XGBClassifier | 0.913333 | 0.915226 | 0.911433 | 0.913325 |
| | | | LightGBM | 0.902367 | 0.904988 | 0.899733 | 0.902354 |
| | | | FFNN | 0.866667 | 0.873824 | 0.8594 | 0.866495 |
| | Distance | AST+BAF+Jimple | RandomForest | 0.9251 | 0.950049 | 0.899133 | 0.923901 |
| | | | RotationForest | 0.9257 | 0.941441 | 0.909 | 0.924936 |
| | | | XGBClassifier | 0.93566667 | 0.948055 | 0.922733 | 0.935218 |
| | | | LightGBM | 0.93186667 | 0.946155 | 0.916867 | 0.931282 |
| | | | FFNN | 0.9154 | 0.922539 | 0.907733 | 0.914992 |
| | | AST | RandomForest | 0.8704 | 0.940907 | 0.795533 | 0.862301 |
| | | | RotationForest | 0.867833 | 0.907325 | 0.822733 | 0.863004 |
| | | | XGBClassifier | 0.881 | 0.915274 | 0.842533 | 0.877424 |
| | | | LightGBM | 0.874033 | 0.919725 | 0.823267 | 0.868882 |
| | | | FFNN | 0.866467 | 0.883022 | 0.846933 | 0.864431 |
| | | BAF | RandomForest | 0.872533 | 0.928558 | 0.811467 | 0.866174 |
| | | | RotationForest | 0.8675 | 0.90114 | 0.828533 | 0.863328 |
| | | | XGBClassifier | 0.873867 | 0.902537 | 0.8408 | 0.87058 |
| | | | LightGBM | 0.865133 | 0.905707 | 0.8186 | 0.860016 |
| | | | FFNN | 0.863367 | 0.884755 | 0.8376 | 0.860513 |
| | | Jimple | RandomForest | 0.911333 | 0.931023 | 0.89 | 0.910049 |
| | | | RotationForest | 0.907033 | 0.924752 | 0.8876 | 0.905785 |
| | | | XGBClassifier | 0.9125 | 0.926855 | 0.8968 | 0.911582 |
| | | | LightGBM | 0.9052 | 0.92326 | 0.885333 | 0.903884 |
| | | | FFNN | 0.8905 | 0.914434 | 0.863733 | 0.888268 |
| | Multiplicative | AST+BAF+Jimple | RandomForest | 0.9528 | 0.965215 | 0.940267 | 0.952577 |
| | | | RotationForest | 0.954767 | 0.963618 | 0.9458 | 0.954624 |
| | | | XGBClassifier | 0.9564 | 0.963844 | 0.948867 | 0.956292 |
| | | | LightGBM | 0.954967 | 0.964701 | 0.945133 | 0.954816 |
| | | | FFNN | 0.895533 | 0.919294 | 0.8744 | 0.894599 |
| | | AST | RandomForest | 0.9416 | 0.963243 | 0.919667 | 0.94096 |
| | | | RotationForest | 0.9415 | 0.958812 | 0.9238 | 0.940981 |
| | | | XGBClassifier | 0.945 | 0.960377 | 0.929333 | 0.944597 |
| | | | LightGBM | 0.9424 | 0.961552 | 0.922933 | 0.941847 |
| | | | FFNN | 0.86301 | 0.947706 | 0.911733 | 0.92935 |
| | | BAF | RandomForest | 0.933333 | 0.951904 | 0.914067 | 0.932592 |
| | | | RotationForest | 0.928967 | 0.942612 | 0.914533 | 0.928355 |
| | | | XGBClassifier | 0.933967 | 0.949961 | 0.917333 | 0.933356 |
| | | | LightGBM | 0.929867 | 0.948916 | 0.91 | 0.929047 |
| | | | FFNN | 0.859467 | 0.930223 | 0.887133 | 0.908031 |
| | | Jimple | RandomForest | 0.937533 | 0.956612 | 0.917933 | 0.936877 |
| | | | RotationForest | 0.939167 | 0.951263 | 0.9266 | 0.938762 |
| | | | XGBClassifier | 0.942233 | 0.956946 | 0.927133 | 0.941801 |
| | | | LightGBM | 0.9381 | 0.95643 | 0.919267 | 0.93748 |
| | | | FFNN | 0.810833 | 0.902168 | 0.709667 | 0.791924 |
| 40,000 | Linear | AST+BAF+Jimple | RandomForest | 0.967143 | 0.96789765 | 0.966389 | 0.967142 |
| | | | RotationForest | 0.967731 | 0.96834034 | 0.967132 | 0.967735 |
| | | | XGBClassifier | 0.967395 | 0.96853364 | 0.966253 | 0.967391 |
| | | | LightGBM | 0.967563 | 0.96844997 | 0.966671 | 0.967559 |
| | | | FFNN | 0.942211 | 0.94438599 | 0.940017 | 0.942192 |
| | | AST | RandomForest | 0.925212 | 0.925839 | 0.924574 | 0.925205 |
| | | | RotationForest | 0.934204 | 0.934784 | 0.933618 | 0.9342 |
| | | | XGBClassifier | 0.937236 | 0.9385 | 0.935968 | 0.937232 |
| | | | LightGBM | 0.941263 | 0.942718 | 0.939796 | 0.941254 |
| | | | FFNN | 0.919082 | 0.920721 | 0.917471 | 0.919079 |
| | | BAF | RandomForest | 0.904961 | 0.906528 | 0.903387 | 0.904955 |
| | | | RotationForest | 0.916925 | 0.918059 | 0.915786 | 0.91692 |
| | | | XGBClassifier | 0.926395 | 0.928211 | 0.924571 | 0.926387 |

| | | | | | | | |
|---|---|---|---|---|---|---|---|
| | | | LightGBM | 0.952524 | 0.954383 | 0.950657 | 0.952515 |
| | | | FFNN | 0.894035 | 0.897068 | 0.89096 | 0.893997 |
| | | Jimple | RandomForest | 0.885017 | 0.886913 | 0.883103 | 0.885003 |
| | | | RotationForest | 0.902729 | 0.903755 | 0.901703 | 0.902726 |
| | | | XGBClassifier | 0.925555 | 0.927565 | 0.923531 | 0.925543 |
| | | | LightGBM | 0.92076 | 0.923435 | 0.918073 | 0.920745 |
| | | | FFNN | 0.872769 | 0.88077 | 0.864577 | 0.872584 |
| | Distance | AST+BAF+Jimple | RandomForest | 0.926717923 | 0.951007 | 0.901512 | 0.925607 |
| | | | RotationForest | 0.926759936 | 0.945049 | 0.907529 | 0.925903 |
| | | | XGBClassifier | 0.937649777 | 0.950424 | 0.924375 | 0.937212 |
| | | | LightGBM | 0.930969666 | 0.946196 | 0.915006 | 0.930333 |
| | | | FFNN | 0.898021175 | 0.910891 | 0.884107 | 0.897284 |
| | | AST | RandomForest | 0.875588 | 0.941804 | 0.805392 | 0.868423 |
| | | | RotationForest | 0.874874 | 0.913211 | 0.831564 | 0.870509 |
| | | | XGBClassifier | 0.8841 | 0.918386 | 0.845886 | 0.880674 |
| | | | LightGBM | 0.874252 | 0.922443 | 0.821051 | 0.868868 |
| | | | FFNN | 0.85042 | 0.870567 | 0.826688 | 0.847963 |
| | | BAF | RandomForest | 0.874512 | 0.932448 | 0.811849 | 0.868091 |
| | | | RotationForest | 0.872571 | 0.907788 | 0.832333 | 0.868449 |
| | | | XGBClassifier | 0.875437 | 0.905535 | 0.840897 | 0.872027 |
| | | | LightGBM | 0.863463 | 0.904479 | 0.816283 | 0.858164 |
| | | | FFNN | 0.843774 | 0.857339 | 0.827439 | 0.841998 |
| | | Jimple | RandomForest | 0.912938 | 0.933559 | 0.890711 | 0.911635 |
| | | | RotationForest | 0.911316 | 0.929592 | 0.891445 | 0.910121 |
| | | | XGBClassifier | 0.914131 | 0.930495 | 0.896345 | 0.913104 |
| | | | LightGBM | 0.906963 | 0.926513 | 0.885553 | 0.905577 |
| | | | FFNN | 0.867923 | 0.894197 | 0.838682 | 0.865349 |
| | Multiplicative | AST+BAF+Jimple | RandomForest | 0.954942 | 0.965674 | 0.944101933 | 0.954765 |
| | | | RotationForest | 0.956749 | 0.963866 | 0.949526315 | 0.95664 |
| | | | XGBClassifier | 0.95806 | 0.965417 | 0.950619472 | 0.95796 |
| | | | LightGBM | 0.956228 | 0.965187 | 0.947184866 | 0.9561 |
| | | | FFNN | 0.93238 | 0.935288 | 0.929480371 | 0.932333 |
| | | AST | RandomForest | 0.941952 | 0.9635 | 0.920118 | 0.941315 |
| | | | RotationForest | 0.942372 | 0.959997 | 0.924392 | 0.941858 |
| | | | XGBClassifier | 0.945027 | 0.959917 | 0.92982 | 0.944628 |
| | | | LightGBM | 0.941759 | 0.961113 | 0.922046 | 0.941178 |
| | | | FFNN | 0.85042 | 0.870567 | 0.826688 | 0.847963 |
| | | BAF | RandomForest | 0.935734 | 0.953155 | 0.917681 | 0.935086 |
| | | | RotationForest | 0.933524 | 0.94603 | 0.92038 | 0.933026 |
| | | | XGBClassifier | 0.93607 | 0.952205 | 0.919292 | 0.935461 |
| | | | LightGBM | 0.930508 | 0.951224 | 0.908957 | 0.929618 |
| | | | FFNN | 0.893719 | 0.909213 | 0.877245 | 0.892793 |
| | | Jimple | RandomForest | 0.938582 | 0.958183 | 0.918492 | 0.93792 |
| | | | RotationForest | 0.935734 | 0.950739 | 0.920106 | 0.93517 |
| | | | XGBClassifier | 0.943372 | 0.956679 | 0.929692 | 0.942989 |
| | | | LightGBM | 0.93844 | 0.956424 | 0.919938 | 0.93783 |
| | | | FFNN | 0.808356 | 0.847771 | 0.763754 | 0.799527 |

## 3. Evaluation of Performance with Different Feature Sizes: Experimental Results

| Combination technique | Feature size | Classifiers | Accuracy | Precision | Recall | F1 |
|---|---|---|---|---|---|---|
| Linear | 20 | RandomForest | 0.9300333 | 0.93113 | 0.928933 | 0.93003 |
| | | RotationForest | 0.9474 | 0.947932 | 0.946867 | 0.947398 |
| | | XGBClassifier | 0.953 | 0.954496 | 0.9515 | 0.952996 |
| | | LightGBM | 0.9570667 | 0.958331 | 0.9558 | 0.957063 |
| | 30 | RandomForest | 0.9313667 | 0.932363 | 0.930367 | 0.931363 |
| | | RotationForest | 0.9472667 | 0.947932 | 0.9466 | 0.947265 |
| | | XGBClassifier | 0.952 | 0.953297 | 0.9507 | 0.951997 |
| | | LightGBM | 0.9583333 | 0.959531 | 0.957133 | 0.958329 |
| | 40 | RandomForest | 0.9164 | 0.937298 | 0.9355 | 0.936397 |
| | | RotationForest | 0.9275333 | 0.958002 | 0.957067 | 0.957533 |
| | | XGBClassifier | 0.9371 | 0.958131 | 0.956067 | 0.957096 |
| | | LightGBM | 0.9396667 | 0.960864 | 0.958467 | 0.959664 |
| | 50 | RandomForest | 0.9510333 | 0.951797 | 0.950267 | 0.951031 |
| | | RotationForest | 0.9565667 | 0.956803 | 0.956333 | 0.956566 |
| | | XGBClassifier | 0.9566667 | 0.957433 | 0.9559 | 0.956664 |
| | | LightGBM | 0.9634667 | 0.964431 | 0.9625 | 0.963464 |
| | 60 | RandomForest | 0.9571 | 0.957866 | 0.956333 | 0.957098 |
| | | RotationForest | 0.9617 | 0.962166 | 0.961233 | 0.961699 |
| | | XGBClassifier | 0.9632667 | 0.964498 | 0.962033 | 0.963264 |
| | | LightGBM | 0.9673333 | 0.968731 | 0.965933 | 0.96733 |
| Distance | 20 | RandomForest | 0.8347 | 0.936878771 | 0.785733333 | 0.854789284 |
| | | RotationForest | 0.847233333 | 0.917791976 | 0.830933333 | 0.872239081 |
| | | XGBClassifier | 0.8518 | 0.913268977 | 0.846733333 | 0.878757195 |
| | | LightGBM | 0.866366667 | 0.943505623 | 0.843933333 | 0.890913813 |
| | 30 | RandomForest | 0.8717 | 0.942732809 | 0.795 | 0.862678704 |
| | | RotationForest | 0.886333333 | 0.92549532 | 0.842466667 | 0.882019935 |
| | | XGBClassifier | 0.895566667 | 0.924515433 | 0.863866667 | 0.893174336 |
| | | LightGBM | 0.907333333 | 0.947853874 | 0.862933333 | 0.903403938 |
| | 40 | RandomForest | 0.917566667 | 0.95559426 | 0.8776 | 0.914953286 |
| | | RotationForest | 0.918266667 | 0.939032112 | 0.896133333 | 0.917088017 |
| | | XGBClassifier | 0.9273 | 0.941639334 | 0.912266667 | 0.926713523 |
| | | LightGBM | 0.932066667 | 0.94929115 | 0.913933333 | 0.931271796 |
| | 50 | RandomForest | 0.9208 | 0.955386358 | 0.884466667 | 0.918561862 |
| | | RotationForest | 0.924033333 | 0.942281679 | 0.904733333 | 0.923123701 |
| | | XGBClassifier | 0.928 | 0.942075271 | 0.913333333 | 0.9274799 |
| | | LightGBM | 0.939533333 | 0.95479399 | 0.923433333 | 0.938846161 |
| | 60 | RandomForest | 0.922866667 | 0.957029826 | 0.887066667 | 0.920725268 |
| | | RotationForest | 0.9293 | 0.946017436 | 0.9116 | 0.928476755 |
| | | XGBClassifier | 0.930766667 | 0.944088255 | 0.916866667 | 0.930270856 |
| | | LightGBM | 0.939366667 | 0.954569385 | 0.9234 | 0.938726047 |
| Multiplicative | 20 | RandomForest | 0.854266667 | 0.899535413 | 0.848466667 | 0.87331476 |
| | | RotationForest | 0.882733333 | 0.948169912 | 0.916933333 | 0.932290842 |
| | | XGBClassifier | 0.9154 | 0.9520649 | 0.918433333 | 0.93495425 |
| | | LightGBM | 0.912566667 | 0.962020224 | 0.9228 | 0.94200974 |
| | 30 | RandomForest | 0.880866667 | 0.930417223 | 0.890933333 | 0.91027194 |
| | | RotationForest | 0.9276 | 0.960958426 | 0.934 | 0.947282995 |
| | | XGBClassifier | 0.951766667 | 0.963521678 | 0.939866667 | 0.951544787 |
| | | LightGBM | 0.949566667 | 0.963013954 | 0.935933333 | 0.949281717 |
| | 40 | RandomForest | 0.946033333 | 0.961944308 | 0.929866667 | 0.945636747 |
| | | RotationForest | 0.944666667 | 0.95389378 | 0.9353 | 0.944500002 |
| | | XGBClassifier | 0.9546 | 0.964021778 | 0.945066667 | 0.954451215 |
| | | LightGBM | 0.9531 | 0.964341899 | 0.941733333 | 0.952902643 |
| | 50 | RandomForest | 0.947633333 | 0.964079615 | 0.931 | 0.947252657 |
| | | RotationForest | 0.949366667 | 0.958112155 | 0.9405 | 0.949220961 |
| | | XGBClassifier | 0.9562 | 0.964841538 | 0.947466667 | 0.95606937 |
| | | LightGBM | 0.954733333 | 0.964564013 | 0.9448 | 0.95457398 |
| | 60 | RandomForest | 0.947633333 | 0.964497812 | 0.931866667 | 0.947900968 |
| | | RotationForest | 0.954833333 | 0.963688022 | 0.945866667 | 0.945866667 |
| | | XGBClassifier | 0.956866667 | 0.965058209 | 0.9486 | 0.9567551 |
| | | LightGBM | 0.955633333 | 0.965108102 | 0.946066667 | 0.955493365 |

## 4. Evaluation of Performance in Semantic Clone Detection: Experiment Results

| Combination Technique | Clone Type | Classifiers | Precision | Recall | F1 |
|---|---|---|---|---|---|
| Linear | VST3 | RandomForest | 91 | 90 | 90 |
| | | RotationForest | 91 | 91 | 91 |
| | | XGBClassifier | 90.6 | 92 | 91 |
| | | LightGBM | 97.9 | 96.2 | 97.04 |
| | | FFNN | 88 | 89 | 89 |
| | ST3 | RandomForest | 84 | 78 | 81 |
| | | RotationForest | 85 | 79 | 82 |
| | | XGBClassifier | 83 | 80 | 82 |
| | | LightGBM | 97.01 | 96.8 | 96.9 |
| | | FFNN | 75 | 70 | 72 |
| | MT3 | RandomForest | 90 | 90 | 90 |
| | | RotationForest | 91 | 91 | 91 |
| | | XGBClassifier | 95 | 91 | 93 |
| | | LightGBM | 96.5 | 96.3 | 96.4 |
| | | FFNN | 93 | 87 | 90 |
| | WT3/4 | RandomForest | 79 | 85 | 82 |
| | | RotationForest | 78.8 | 85 | 82 |
| | | XGBClassifier | 81 | 86 | 83 |
| | | LightGBM | 95 | 89.7 | 92.3 |
| | | FFNN | 71 | 80 | 75 |
| Distance | VST3 | RandomForest | 77 | 93 | 84.24 |
| | | RotationForest | 81.7 | 93 | 86.98 |
| | | XGBClassifier | 77 | 93 | 84.24 |
| | | LightGBM | 77 | 93 | 84.24 |
| | | FFNN | 74 | 92 | 82.02 |
| | ST3 | RandomForest | 83 | 63 | 71.63 |
| | | RotationForest | 82 | 65 | 72.51 |
| | | XGBClassifier | 81 | 66 | 72.73 |
| | | LightGBM | 80 | 66 | 72.32 |
| | | FFNN | 68 | 63 | 65.40 |
| | MT3 | RandomForest | 89 | 87 | 87.98 |
| | | RotationForest | 90 | 89 | 89.49 |
| | | XGBClassifier | 91 | 89 | 89.98 |
| | | LightGBM | 92 | 89 | 90.47 |
| | | FFNN | 91 | 89 | 89.98 |
| | WT3/4 | RandomForest | 75 | 83 | 78.79 |
| | | RotationForest | 77 | 84 | 80.34 |
| | | XGBClassifier | 78 | 82 | 79.95 |
| | | LightGBM | 79 | 83 | 80.95 |
| | | FFNN | 79 | 72 | 75.33 |
| Multiplicative | VST3 | RandomForest | 90 | 89 | 89.49 |
| | | RotationForest | 90 | 89 | 89.49 |
| | | XGBClassifier | 89 | 91 | 89.98 |
| | | LightGBM | 90 | 91 | 90.49 |
| | | FFNN | 79 | 87 | 82.80 |
| | ST3 | RandomForest | 85 | 79 | 81.8902439 |
| | | RotationForest | 85 | 80 | 82.42424242 |
| | | XGBClassifier | 83 | 81 | 81.9878049 |
| | | LightGBM | 84 | 81 | 82.4727273 |
| | | FFNN | 70 | 55 | 61.6 |
| | MT3 | RandomForest | 87 | 90 | 88.47457627 |
| | | RotationForest | 87 | 91 | 88.95505618 |
| | | XGBClassifier | 91 | 91 | 91 |
| | | LightGBM | 92 | 91 | 91.4972678 |
| | | FFNN | 90 | 84 | 86.89655172 |
| | WT3/4 | RandomForest | 78 | 85 | 81.34969325 |
| | | RotationForest | 79 | 85 | 81.8902439 |
| | | XGBClassifier | 81 | 85 | 82.9518072 |
| | | LightGBM | 80 | 86 | 82.8915663 |
| | | FFNN | 64 | 79 | 70.71328671 |
