# Supplementary material for: A novel code representation for detecting Java code clones using high-level and abstract compiled code representations
Source: PLoS One. 2024 May 10;19(5):e0302333. doi: 10.1371/journal.pone.0302333 (PMC11086904; doi:10.1371/journal.pone.0302333)
Supplement: Attachment — Submitted filename: Response to Reviewers.docx [file pone.0302333.s005.docx]

I would like to express my sincere gratitude to the Editor and the reviewers. Your valuable feedback and insights are greatly appreciated, and we are committed to addressing all your comments and suggestions to enhance the quality and impact of our work.

1. **Editor's Journal comments and responses**

- **Comment 1**: [*Please ensure that your manuscript meets PLOS ONE's style requirements, including those for file naming.*]

**Response** : We would like to inform you that we have adhered to PLOS ONE's style requirements as outlined in the PLOS ONE style templates.

- **Comment 2**: [*Please note that PLOS ONE has specific guidelines on code sharing for submissions in which author-generated code underpins the findings in the manuscript. In these cases, all author-generated code must be made available without restrictions upon publication of the work.***]**

**Response** : Thank you for your concerns regarding the  PLOS ONE guidelines on code sharing requirement. We assure you that we are fully committed to transparency and are willing to provide any details about the source code to reviewers upon request.

- **Comment 3**: [*Please provide a complete Data Availability Statement in the submission form, ensuring you include all necessary access information or a reason for why you are unable to make your data freely accessible. If your research concerns only data provided within your submission, please write "All data are in the manuscript and/or supporting information files" as your Data Availability Statement*.]

**Response** : In reference to the Data Availability statement, all data are presently enclosed within our paper. Furthermore, a supporting information files has been included, encompassing all results we obtained and the datasets that we used in the experiments. Consequently, I respectfully request a modification of the Data Availability statement to state, " The dataset utilized in the present study can be accessed at the following link: <https://github.com/clonebench/BigCloneBench?tab=readme-ov-file> and support file S4"

2. **Comments from Reviewer 1 and Responses**

- **Comment 1:** **[***Increased complexity: Combining multiple code representations adds complexity over single-representation techniques.]*

**Response :** We appreciate the insightful suggestion from the reviewer regarding the increased complexity associated with combining multiple code representations. While it's true that integrating multiple representations may introduce additional complexity, it is essential to weigh this against the benefits gained in terms of enhanced accuracy and robustness in clone detection.

When considering the complexities involved in code clone detection, it is crucial to note that researchers commonly categorize code clones into four types: Type-I, Type-II, Type-III, and Type-IV. The first three rely on textual similarities, whereas Type-IV focuses more on functional or semantic resemblances, posing challenges for detection based solely on textual similarities.

Traditionally, techniques have concentrated on detecting Types I-III clones, employing single code representations like sequences of tokens, trees, graphs, or software metrics. While successful for these types, they often struggle with Type-IV clones due to lexical differences but similar functionality. To overcome this challenge, hybrid code representation has emerged as a promising approach, integrating multiple techniques such as text, tokens, AST, PDG, and metrics. This comprehensive approach enhances clone detection accuracy by capturing semantic similarities that are difficult to detect using a single representation [1-3].

Ultimately, the trade-off between complexity and benefits should be carefully considered. We believe that the advantages gained in terms of improved clone detection and classification, while reducing false positives, outweigh the increased complexity introduced by combining multiple code representations.

- **Comment 2: [***Scalability issues: Deriving and analyzing multiple representations could hamper analysis of very large codebases.***]**

**Response :** We appreciate the insightful recommendation from the reviewer. It would indeed be interesting to explore scalability issues associated with deriving and analyzing multiple code representations. However, our main objective in this work is to propose a new code representation clones and evaluate its effectiveness in detecting both syntactic and semantic clones. While scalability analysis was not within the scope of this work, this approach aligns with the methodology applied in other studies in this domain [2] [4-7].

Nonetheless, we acknowledge that this comment has highlighted an opportunity for future and more extensive research in this direction. We have incorporated a recommendation to explore this opportunity in the revised manuscript. Please refer to page 32 of the revised manuscript, lines 741–743.

- **Comment 3:** [*Sensitivity to changes: Small code modifications may invalidate code representations, requiring re-analysis.***]**

**Response :** We are grateful for the reviewer's insightful suggestion, and we acknowledge the importance of sensitivity to changes. In response to this valuable comment, we want to clarify how we made the proposed technique robust for source code changes.

Firstly, in the preprocessing stage several normalization operations have been applied, including the removal of unnecessary white spaces, the exclusion of comments, and the substitution of variable names and function names with generic placeholders to mitigate disparities in naming conventions. Numeric literals, string literals, and other constants have with placeholders to generalize specific values. For detailed information, please refer to the revised manuscript, specifically on page 11, lines 266–273.

Secondly, the Soot Framework, a static analysis tool, has been used to optimize and represent Java source code by carrying out transformations such as eliminating repetitious code, dead code elimination, unused local variables elimination, and common sub-expression elimination. This significantly reduced the number of operations required to represent the java source code. The soot framework provides four intermediate representations (IRs). Each of the IRs has different levels of abstraction that provide several benefits when analyzing and transforming java code. Further details can be found on pages 5-8, lines 126-194.

- **Comment 4:** [*Variability across languages: Representations and extracted features may not generalize well across different programming languages*.**]**

**Response :** Thank you for raising the concern about the variability across languages in the generalization of representations and extracted features. While it's true that representations and features may not generalize well across different programming languages, it's important to note that our proposed code representation is specifically designed and optimized for detecting clones in Java code.

We acknowledge that variability across languages can pose challenges in generalization, and we have focused our efforts on developing a representation that is tailored to the characteristics of Java code. By leveraging domain-specific knowledge and utilizing techniques that are specific to Java programming, we aim to maximize the effectiveness of our representation in detecting clones within the Java ecosystem.

While our representation may not directly generalize to other programming languages, we believe that the principles and methodologies employed in its development can serve as valuable insights for researchers working on clone detection in other languages. Additionally, future research could explore the adaptation of our techniques to other languages, taking into account their unique characteristics and requirements.

We have acknowledged this limitation in the threats to validity and limitations section and future work part in the conclusion. Please refer to page 31 of the revised manuscript, lines 715–719 and pages 31 and 32, lines 739-741.

- **Comment 5:** [*Infrastructure requirements: Extraction and processing of representations requires language-specific tool chains/infrastructure.***]**

**Response :** Thank you for bringing up the concern regarding infrastructure requirements for the extraction and processing of representations, which necessitate language-specific toolchains and infrastructure.

In our work, we have developed a novel code representation specifically tailored for detecting clones in Java code. To address these infrastructure requirements, we carefully selected and utilized language-specific toolchains and infrastructure. For example, we used the Soot framework [8] for static analysis and code transformation in Java, the JavaParser [9] tool to build AST and traverse its nodes, and the Stubber [10] tool for compiling Java source code into Bytecode without dependencies. The roles of each tool are specified in the methodology section of the revised manuscript, which can be found on pages 10-20 and in the background section on pages 4-8.

- **Comment 6:** [*Evaluation limitations: Approach tested on limited datasets, more rigorous validation on real-world projects needed.]*

**Response:** We appreciate the reviewer for pointing this out. In this work, we used the BigCloneBench [11] dataset (a real-world dataset), which is a widely used benchmark for assessing Java code clone detection systems. It contains 55,499 Java source files from 24,557 distinct open-source projects, collected through the mining process of IJaDataset-2.0. The current version of this benchmark includes over 8.5 million labelled true clone pairs and more than 260,000 labelled false clone pairs across 43 functionalities, categorized into Type-I, Type-II, Type-III, and Type-IV. State–of–the–art techniques [2, 5, 6, 12] in the field of code clone detection have used this dataset. For more details please refer to the revised manuscript on pages 20-21, lines 433- 460.

- **Comment 7:** *[False positives risks: Semantic abstraction could wrongly link syntactically different code snippets.]*

**Response:** We appreciate the insightful suggestion from the reviewer regarding the risk of false positive. As we know, representing source code in a more abstract manner may lead to extracting more comprehensive semantic features, thereby improving the effectiveness of clone detection techniques in identifying semantic clones. However, the main issue associated with abstract representation is the potential increase in false positive. To mitigate this issue, we employed both high-level source code and abstract low-level compiled code representations in our work. This approach allowed us to leverage the benefits of abstract representation while considering the specific details of the source code. For more details, please refer to the methodology section of the revised manuscript, available on pages 10-20.

- **Comment 8:** *[Configuration challenges: Proper configuration of machine learning models requires hyperparameter tuning expertise.]*

**Response:** Thank you for your interesting comment. In our study, we opted to use the default settings for machine learning models, as specified in the techniques documentation. While hyperparameter tuning can indeed enhance model performance, we chose to adhere to the default settings to maintain consistency and align with established practices outlined in the documentation. By using default settings, we aimed to ensure transparency and reproducibility in our methodology. We acknowledge that further exploration of hyperparameter tuning could be beneficial and may be considered in future research.

- **Comment 9:** *[Explainability challenges: Identifying why certain code is flagged as clones from multiple blended representations is difficult.]*

**Response**: We appreciate the reviewer's insightful comment regarding the challenges of explainability when using multiple blended representations for clone detection. In our work, we recognize the importance of explainability in clone detection and have taken steps to address this challenge. Firstly, we have thoroughly documented our methodology for deriving and analyzing multiple representations. This documentation includes detailed explanations of each representation technique used and how they contribute to clone detection.

Furthermore, we have conducted two experiments to investigate and illustrate how each representation contributes to the identification of code clones. In the first experiment, we evaluated the performance of the proposed techniques with different dataset sizes and feature types (AST, Baf, JimplePDG, and AST+Baf+JimplePDG). Please refer to page 23, lines 504-524, in the revised manuscript for more details. To further investigate the importance of the proposed features (code representation) in enhancing performance in code clone detection and to ensure that this improvement is not coincidental, another experiment was conducted involving varying numbers of features, combined by three combination approaches (linear, multiplicative, distance). An equal number of features was selected from each type (AST, BAF, and Jimple PDG). Please refer to page 24,lines 525-538, for more details.

- **Comment 10:** *[Intellectual property risks: Extracting and sharing representations and models could inadvertently leak proprietary code.]*

**Response**: We appreciate the reviewer for highlighting the concern regarding intellectual property risks associated with extracting and sharing representations and models, which could potentially lead to the inadvertent leakage of proprietary code.

To address this concern, we have implemented several precautions in our work. Firstly, we have ensured that all code representations and models used in our study are derived from publicly available and open-source datasets, such as BigCloneBench, which contains labeled clone pairs from various open-source projects. Additionally, both the Soot, Stubber, and the JavaParser tool are freely available for use under the terms of the GNU Lesser General Public License (LGPL) version 2.1 and Eclipse Public License 2.0. Furthermore, we have meticulously anonymized and sanitized any code snippets used in our analysis to remove any proprietary or sensitive information. Overall, we are committed to upholding the highest standards of ethical conduct and data privacy in our research endeavors.

- **Comment 11:** *[Describe dataset features in more details and its total size and size of (train/test) as a table]*

**Response**: Thank you for your valuable feedback. We appreciate your suggestion to provide more detailed information about the dataset features and its total size, as well as the size of the train and test sets, in a table format.

In response to your suggestion, we have included a support file, labeled as S1, which contains detailed information about different types of feature used in this work.

Regarding the dataset and its size, you can refer to Table 5 in the revised manuscript on page 21, which provides an overview of the constructed dataset. For more detailed information, please refer to support file labeled S2, which contains comprehensive details about both the BigCloneBench dataset and the constructed dataset.

As for the size of the training and test sets, we specified the dataset size used in each experiment, please refer to the evaluation section in the revised manuscript on pages 21-27. Additionally, we utilized 10-fold Stratified cross-validation for training and testing our models. This approach ensures robustness and reliability in our models evaluation process by systematically dividing the dataset into training and testing subsets while maintaining the distribution of classes.

We believe that these enhancements offer a more comprehensive understanding of the dataset used in our work and its characteristics.

- **Comment 12:** *[Flowchart and algorithm steps need to be inserted]*

**Response**: We greatly appreciate your recommendation to include flowcharts to illustrate the architecture and algorithms used in our methodology.

We are pleased to inform you that we have incorporated a detailed architecture diagram in the revised manuscript, located in Figure 8 on page 11. This diagram provides a comprehensive overview of our methodology.

Additionally, in response to your suggestion, we have introduced a new figure (Figure 9) on page 11 to illustrate the process of extracting syntactic and semantic features.

Furthermore, we have included pseudo code for each algorithm used in our work, along with detailed explanations of each step within these algorithms. These can be found in the Algorithms section of the revised manuscript on pages 12, 14, 16, and 17.

We believe that these enhancements significantly contribute to the clarity and understanding of our methodology by providing both visual representations of the architecture and detailed explanations of the algorithms.

- **Comment 14:** *[Limitation Section need to be inserted.]*

**Response**: Thank you for bringing this our attention, and we have taken actions to address your comment. In the revised manuscript, we have added a threats to validity and limitations section that contains provides detailed information about the potential threats and limitations of our work. For detailed information, we kindly direct your attention to the revised manuscript, specifically on pages 30 and 31, lines 7699–719.

- **Comment 15:** *[All metrics need to be calculated such as Accuracy, Precision, Recall, F1 score, and ROC AUC score in the experimental results as tables.]*

**Response:** Thank you for your valuable feedback. We appreciate your suggestion to include a comprehensive set of performance metrics, including Accuracy, Precision, Recall, F1 score, and ROC AUC score, in the experimental results.

In response to your comment, we would like to clarify that we have indeed computed all performance metrics, including Accuracy, Precision, Recall, and F1 score, for each experiment conducted in our study. However, due to space constraints and to maintain focus on the main findings of each experiment, we chose to present specific metrics in each experiment.

For Experiment 1, 2, and 3, we presented Accuracy as the primary metric, as these experiments primarily focused on assessing overall classification performance. For Experiment 4, we focused on Recall, Precision, and F1-score, as these metrics are particularly relevant for evaluating the performance of the model in correctly identifying semantic clones (VST3, ST3, MT3, and WT3/4). Similarly, in Experiment 5, we presented Recall and F1-score, as these metrics are essential for comparing the model's performance against other detection techniques.

However, to provide transparency and ensure the availability of all computed metrics, we have included a support file containing the computed metrics (Accuracy, Precision, Recall, and F1-score) for each experiment. This supplementary information allows readers to access a comprehensive set of performance metrics for a more thorough understanding of the experimental results.

We believe that this approach strikes a balance between presenting key findings and ensuring the availability of all relevant metrics for transparency and reproducibility purposes.

- **Comment 16:** *[Address the accuracy/improvement percentages in the abstract and in the conclusion sections, as well as the significance of these results.]*

**Response**: Thank you for bringing this to our attention. We fully agree with your comment, and we have taken action to address your concerns. A detailed discussion section in the revised manuscript analyzes the results obtained in our experiments. Additionally, we have highlighted the significance of these results in both the conclusion and abstract to provide a comprehensive overview of our findings. Please refer to the discussion section, as well as both the conclusion and abstract, in the revised manuscript.

- **Comment 17:** *[The architecture of the proposed model must be provided]*

**Response:** Thank you for pointing this out. We have taken the necessary steps to address your concerns. The architecture of the proposed work is now presented in Figure 8, which can be found in the revised manuscript on page 11.

- **Comment 18:** *[The authors need to make a clear proofread to avoid grammatical mistakes and typo errors.]*

**Response:** Thank you for your feedback. To address your concerns, we conducted a thorough proofreading of the manuscript to rectify any grammatical mistakes and typographical errors.

- **Comment 19:** *[Add future work in last section (conclusion) (if any).]*

**Response:** Thank you for your suggestion. We have taken the necessary steps to address your concerns by incorporating the future work into the last section (conclusion) of the revised manuscript. For detailed information, please refer to pages 31 and 32, lines 734-743 of the revised manuscript.

- **Comment 20:** *[To improve the Related Work and Introduction sections authors are recommended to review this highly related research work paper]*

**Response**: Thank you for your valuable feedback and recommendation. We have thoroughly reviewed the highly related research papers, along with others in the field. These papers have provided an enhanced overview of the existing literature in this domain. As a result, we have incorporated them into the Related Work section on page 9, lines 214-225 of the revised manuscript, and into the subsection on individual machine learning classifiers on page 20, lines 423 and 424, thereby strengthening the discussion on machine learning approaches.

Moreover, we have acknowledged the insights gained from works 1 and 2 in the Discussion section on page 29, lines 664-670 of the revised manuscript, where we highlight potential extensions and future research directions inspired by these studies.

We believe that these updates significantly enrich the Related Work section and provide a more comprehensive understanding of the research landscape and the contributions of our work. Thank you for your suggestion, which has contributed to the overall improvement of our manuscript.

3. **Comments from Reviewer 2 and Responses:**

Comments : [*The paper attempts to report work on using AST to detect Java clones. As the motivation of the work is not clearly defined, the methodology presented is not sound and proven. There is no data, experiment or lemma/theorem to support the proposal. Therefore, the paper is recommended for rejection*.]

Thank you for your valuable feedback. We would like to clarify that our proposed work goes beyond the use of Abstract Syntax Trees (AST) alone to detect code clones; it incorporates other low-level intermediate representations to capture a more comprehensive set of semantic features. Regarding your concerns about the motivation, methodology, and lack of supporting data, experiments, or theorems: we appreciate your insights and have taken significant steps to address these concerns and enhance the quality of our manuscript. Here are our responses to the specific points raised::

1. Experimental Evaluation Addition: We have incorporated new sections dedicated to experimental evaluation to provide a comprehensive understanding of the effectiveness of our proposed technique. Through various experiments, we have explored different aspects of our approach and visually presented our findings using figures.
2. Enhancements to Related Work: Additional literature has been integrated into the related work section, and a table summarizing code representation techniques used in the literature.
3. Dataset Description: A subsection detailing the dataset used in our work has been included within the Experimental Evaluation section. This provides insights into the dataset's characteristics and its relevance to our study.
4. Additions of Threats to Validity and Limitations Section: We have incorporated a dedicated section discussing potential threats to validity and limitations of our study, providing a thorough analysis of these aspects.
5. Updated Discussion, Abstract, and Conclusion Sections : The discussion, abstract, and conclusion sections have been thoroughly revised to offer a more comprehensive analysis of our findings from the experiments.

We believe that these revisions significantly enhance the quality and rigor of our manuscript, addressing your concerns effectively. Thank you for your valuable feedback.

4. **Comments from Reviewer 3 and Responses:**

- **Comment 1***: [Title: it is recommended to rephrase the title to show the approach of clone detection, for example using bytecode or assembly.]*

**Response:**

Thank you for your valuable feedback. We appreciate your suggestion to update the title of the manuscript to better reflect the contribution of our work. We agree that a clear and accurate title is essential for effectively communicating the content and significance of the research. In response to your recommendation, we have revised the title to ensure that it accurately represents the contributions made in the manuscript. The revised title now reads: "A Novel Code Representation for Detecting Java Code Clones Using High-level and Abstract Compiled Code Representations." Your input is greatly appreciated.

- **Comment 2:** *[Abstract: lines from 16 onwards might be too detailed for the abstract. it is recommended to remove them. The issue could be maintainability, reusability and even ethical consideration*.*]*

**Response:** Thank you for pointing this out. In response to your recommendation lines from 16 onwards have been deleted and the abstract section have been updated to reflect our experiments and findings.

- **Comment 3:** *[it is recommended to provide either a table or a tree structure showing subtypes.]*

**Response:** Thank you for bringing this to our attention. We have taken steps to address your recommendation by creating a table that summarizes and compares the code representation techniques used in the literature. Please refer to Table 1 on page 10 of the revised manuscript.

- **Comment 4:** *[The role or type of machine learning used in this study is not clear. This also should reflect the abstract to include the idea of machine learning.]*

**Response:** Thank you for pointing this to out. We would like to clarify that code clone detection techniques typically involve three key steps: (1) Code pre-processing, which eliminates uninteresting items such as header files and comments; (2) Code representation, where source code is transformed into an intermediate representation (e.g., AST, token sequence, or PDG); and (3) Code similarity comparison, where the similarity between code fragments is computed, leading to the identification of code clones when this similarity exceeds a predefined threshold.

In this work, we proposed a new code representation for Java source code that enables code similarity comparison techniques to detect semantic clones. The role of trained machine learning classifiers in this work is to predict the similarity between code fragments and identify code clones, as clearly mentioned in our contribution section on page 4, lines 82-84 of the revised manuscript. Furthermore, we have provided detailed information about the classifiers used in this work. Please refer to pages 19 and 20, lines 393-426 in the revised manuscript for further details.

- **Comment 4:** *[94-100 seems to be repeated. Many parts in the introduction are repeated and define the acronyms on first use.]*

**Response:** Thank you for pointing that out. In response to your recommendation, we have taken the following action: we have rephrased lines 92-101 and removed repeated parts to better reflect our contribution. Furthermore, we have checked the manuscript and correct the use of acronyms.

- **Comment 5:** *[the background should come before related works..]*

**Response:** Thank you for your feedback. We appreciate your suggestion. In response, we have reorganized the manuscript to move the background section before the related works section. This change aligns with the standard structure and enhances the flow of the paper. You can now find the background section preceding the related works section in the revised manuscript.

- **Comment 6:** *[instead of saying, "will be normalized and compiled using a compilation tool like the Stub", authors should specify what they use and why.]*

**Response:** Thank you for your valuable feedback. We have addressed this concern by providing more details about the normalization operations and specifying the tools used for both normalization and compilation. Specifically, we clarified that the files undergo normalization operations such as the removal of unnecessary white spaces and comments. Additionally, we explicitly mentioned that the Stubber tool is employed for both normalizing and compiling the files. We selected this tool for its efficiency and compatibility with our dataset. For further elaboration on these processes, please refer to the methodology section in the revised manuscript, page 11, lines 265-273.

- **Comment 7:** *[Step 3 is not detailed at all, and it is suggested to be removed.]*

**Response:** Thank you for your valuable feedback. We acknowledge your suggestion regarding Step 3 and have carefully considered it. However, we believe that Step 3, which involves the machine learning stage, is crucial for the overall effectiveness of our approach.

In traditional code clone detection techniques, comparison algorithms such as string matching, tree-based matching, and sub-graph matching are commonly used to compare transformed code fragments with each other to identify similar code fragments or clones. In our work, we have taken a different approach by utilizing machine learning classifiers to perform this task. These classifiers are trained on the transformed code representations generated in Step 2 and are capable of effectively detecting semantic clones.

The inclusion of the machine learning stage allows us to leverage the power of various classification algorithms, including ensemble and individual techniques, to evaluate their effectiveness in detecting semantic clones using the proposed features. This approach not only enhances the accuracy of clone detection but also provides flexibility and scalability in handling complex codebases.

While we understand your suggestion to remove Step 3 for brevity, we believe that detailing this crucial stage adds depth to our methodology and contributes to a better understanding of our approach. We have ensured that the methodology section provides comprehensive information about each stage of our process, including Step 3. Please refer to pages 19 and 20 in the revised manuscript for more details.

- **Comment 8:** *[there are no results of this study, so no generalization can be made.]*

**Response:** Thank you for your comment. We appreciate your concern regarding the absence of results in our study. We would like to clarify that extensive experimental work has indeed been conducted to evaluate the effectiveness of the proposed techniques and to provide insights into various perspectives regarding the proposed representation.

In our revised manuscript, we have included a dedicated section titled "Experimental Evaluation" (pages 20-27) where we present detailed results of the experiments conducted. These experiments cover a range of scenarios and settings to thoroughly evaluate the performance and effectiveness of our proposed techniques.

We have rigorously analyzed the experimental results and provided comprehensive discussions on the findings, including the strengths, limitations, and implications of our approach. Through this thorough evaluation, we aim to provide valuable insights and contribute to the advancement of knowledge in the field of code clone detection.

We believe that the inclusion of these experimental results and analyses strengthens the validity and generalizability of our study.

- **Comment 9:** *[no limitations or dataset description.]*

**Response:** Thank you for your feedback. We appreciate your attention to the important aspects of our study, including the limitations and dataset description. In response to your comments, we have made the following enhancements to our manuscript:

1- Threats to Validity and Limitations Section: We have added a dedicated section titled "Threats to Validity and Limitations" in the revised manuscript. This section provides a detailed discussion of potential threats to the validity of our study and acknowledges the limitations inherent in our approach. You can find this section on pages 30 and 31, lines 699-719.
2- Dataset Description: Additionally, we have included a subsection within the "Experimental Evaluation" section to describe the dataset used in our work. This description provides insights into the characteristics of the dataset and its relevance to our study. You can refer to pages 20 and 21, lines 433-460, for detailed information about the dataset.

We believe that these additions significantly improve the comprehensiveness and transparency of our manuscript by addressing your concerns regarding limitations and dataset description.

- **Comment 10:** *[It is highly recommended to have a section for used datasets and tools]*

**Response:** Thank you for your valuable recommendation. We have addressed this concern by providing more detailed information about the tools used in our work throughout the manuscript. In the background section, we discussed the Soot framework and its intermediate representations on pages 5-8, lines 126-194, and the JavaParser tool for generating and processing ASTs on page 5, lines 118-125. Additionally, we described the Stubber tool in the methodology subsection on page 11. Furthermore, we included information about the dataset used in the experimental evaluation, as mentioned in our response to comment 9. Therefore, we believe that we have adequately addressed the details of the datasets and tools used in our work.

- **Comment 11***:[Discussion is shallow, focus on research questions and the objectives, please]*

**Response:** Thank you for your feedback and for highlighting the importance of a comprehensive discussion that addresses the research questions and objectives. In response to your comment, we have thoroughly revised the discussion section of our manuscript to ensure a deeper analysis for our finding in the experiments.

We believe that these updates have enhanced the depth and relevance of the discussion section, aligning it more closely with the objectives of our study. Please refer to the revised manuscript, particularly the discussion section on page 27-30, for a comprehensive analysis based on the experimental findings and research objectives.

- **Comment 12***:[conclusion: it looks like a summary, please revise and implications, limitations, and threats are crucial sections.]*

**Response:** Thank you for your feedback and for emphasizing the importance of including implications, limitations, and threats to validity sections in the manuscript. We have carefully revised the conclusion section to ensure that it goes beyond a mere summary and adequately addresses these crucial aspects. Please refer to pages 31 and 32 on the revised manuscript.

Furthermore, we have introduced a dedicated section on threats to validity and limitations , where we discuss various factors that could affect the reliability and generalizability of our results. This section aims to provide transparency and ensure the rigor of our research methodology. Please refer to pages 30 and 31 on the revised manuscript.

We believe that these updates have strengthened the conclusion section, making it more comprehensive and reflective of the key findings and contributions of our study.

**Comment 13*:****[Minorissues*

1. *line 28- However, this approach ==>However, in this approach*
2. *, l section numbering and figures are not inserted in their place.*
3. *all figures are blur.]*

**Response** : Thank you for your feedback. We have taken the following action:

1- We rephrase line 28 to be " However, this practice often leads to the emergence of similar or identical code fragments within the software system, typically termed code clones." Please refer to page 2, lines 23 and 24 on the revised manuscript.
2- We have addressed the issue of numbering section by adding numbering to the sections in the manuscript. Now, readers can easily navigate through the document using the section numbers provided.
3- We followed the guidelines provided by the journal, which specify that figures should only be captioned without being inserted into their places within the manuscript. We have ensured that all figures are appropriately labeled and placed according to the journal's requirements.
4- We apologize for any inconvenience caused. When submitting your paper to PLoS ONE, each file must be uploaded individually, as the system compiles a merged PDF from these files. Consequently, figures may appear blurry. However, this is a normal occurrence, and it does not pose an issue as the academic editor and reviewers will still access the higher-resolution source files. It's important to note that only the higher-quality figures will be published, not the blurry ones. For further guidance, please refer to the following link: https://everyone.plos.org/2011/02/11/ask-everyone-figure-files-in-your-merged-pdf/

**References**

1.	Roopam, Singh G, editors. To enhance the code clone detection algorithm by using hybrid approach for detection of code clones. 2017 International Conference on Intelligent Computing and Control Systems (ICICCS); 2017 15-16 June 2017.

2.	Hua W, Sui Y, Wan Y, Liu G, Xu G. FCCA: Hybrid Code Representation for Functional Clone Detection Using Attention Networks. IEEE Transactions on Reliability. 2021;70(1):304-18.

3.	Mehrotra N, Sharma A, Jindal A, Purandare R. Improving Cross-Language Code Clone Detection via Code Representation Learning and Graph Neural Networks. IEEE Transactions on Software Engineering. 2023;49(11):4846-68.

4.	Fang C, Liu Z, Shi Y, Huang J, Shi Q, editors. Functional code clone detection with syntax and semantics fusion learning. Proceedings of the 29th ACM SIGSOFT International Symposium on Software Testing and Analysis; 2020.

5.	Zhang J, Wang X, Zhang H, Sun H, Wang K, Liu X, editors. A novel neural source code representation based on abstract syntax tree. 2019 IEEE/ACM 41st International Conference on Software Engineering (ICSE); 2019: IEEE.

6.	Sheneamer A, Roy S, Kalita J. An Effective Semantic Code Clone Detection Framework Using Pairwise Feature Fusion. IEEE Access. 2021;9:84828-44.

7.	Sheneamer AM. Multiple Similarity-based Features Blending for Detecting Code Clones using Consensus-Driven Classification. Expert Systems with Applications. 2021;183:115364.

8.	Vallée-Rai R, Co P, Gagnon E, Hendren L, Lam P, Sundaresan V. Soot: a Java bytecode optimization framework. 2010 CASCON First Decade High Impact Papers: IBM Corp. p. 214–24.

9.	Smith N, Van Bruggen D, Tomassetti FJL, oct. de. Javaparser: visited. 2017.

10.	Schäfer A, Amme W, Heinze TS, editors. Stubber: Compiling Source Code into Bytecode without Dependencies for Java Code Clone Detection. IEEE 15th International Workshop on Software Clones (IWSC); 2021 2-2 Oct. 2021.

11.	Svajlenko J, Roy CK, editors. Evaluating clone detection tools with BigCloneBench. 2015 IEEE International Conference on Software Maintenance and Evolution (ICSME); 2015 29 Sept.-1 Oct. 2015.

12.	Saini V, Farmahinifarahani F, Lu Y, Baldi P, Lopes CV, editors. Oreo: Detection of clones in the twilight zone. Proceedings of the 26th ACM Joint Meeting on European Software Engineering Conference and Symposium on the Foundations of Software Engineering; 2018.
